# Intra-Airway Treatment with Synthetic Lipoxin A4 and Resolvin E2 Mitigates Neonatal Asthma Triggered by Maternal Exposure to Environmental Particles

**DOI:** 10.3390/ijms24076145

**Published:** 2023-03-24

**Authors:** Mohankumar Ramar, Naohiro Yano, Alexey V. Fedulov

**Affiliations:** Division of Surgical Research, Department of Surgery, Rhode Island Hospital, Alpert Medical School of Brown University, 593 Eddy Street, Providence, RI 02903, USA

**Keywords:** asthma, maternal transmission of asthma, concentrated urban air particles (CAPs), diesel exhaust particles (DEPs), Lipoxin A4, Resolvin E2

## Abstract

Particulate matter in the air exacerbates airway inflammation (AI) in asthma; moreover, prenatal exposure to concentrated urban air particles (CAPs) and diesel exhaust particles (DEPs) predisposes the offspring to asthma and worsens the resolution of AI in response to allergens. We previously tested the hypothesis that such exposure impairs the pathways of specialized proresolving mediators that are critical for resolution and found declined Lipoxin A4 (LxA4) and Resolvin E2 (RvE2) levels in the “at-risk” pups of exposed mothers. Here, we hypothesized that supplementation with synthetic LxA4 or RvE2 via the airway can ameliorate AI after allergen exposure, which has not been tested in models with environmental toxicant triggers. BALB/c newborns with an asthma predisposition resultant from prenatal exposure to CAPs and DEPs were treated once daily for 3 days with 750 ng/mouse of LxA4 or 300 ng/mouse of RvE2 through intranasal instillation, and they were tested with the intentionally low-dose ovalbumin protocol that elicits asthma in the offspring of particle-exposed mothers but not control mothers, mimicking the enigmatic maternal transmission of asthma seen in humans. LxA4 and RvE2 ameliorated the asthma phenotype and improved AI resolution, which was seen as declining airway eosinophilia, lung tissue infiltration, and proallergic cytokine levels.

## 1. Introduction

Asthma is a chronic inflammatory disease that can be triggered or exacerbated by environmental factors including environmental particulate matter (PM). There is a link between air pollution and allergic airway inflammation in individuals who live in cities, heavy-traffic areas, and other places where human exposure to PM is high [1,2,3,4,5,6,7,8,9,10,11,12]. Fine particulate matter (PM2.5) is one of the key environmental pollution components linked in epidemiological and mechanistic studies to asthma onset and the severity of exacerbations [13,14,15,16,17,18]. Not only direct exposure but also prenatal (maternal) exposure to environmental toxicants, including PM, has been linked to asthma and allergic sensitization [19,20,21,22,23,24,25], but there is a lack of consensus on the mechanism.

Our unique model, recapitulating the observations in humans, links PM and asthma: the airway exposure of pregnant mice to PM (concentrated urban air particles (CAPs) and diesel exhaust particles (DEPs)) “predisposes” the offspring to respond to otherwise innocuous low dose of allergen with the asthma phenotype [26,27]. This effect of particles is similar to the effect of maternal allergy [28,29,30,31], and the mechanism of this maternal transmission is not well understood but excludes the “common suspects” of genetic or mitochondrial inheritance, the transplacental transmission of allergens or antibodies (the effect is allergen-independent), etc. Hence, there is a continued need to better understand how PM affects asthma origin and the resolution of asthmatic airway inflammation (AI) because the causality mechanisms between PM and asthma are not clearly established [32].

Specialized proresolving mediators (SPMs) are a class of signaling molecules formed through the metabolism of polyunsaturated fatty acids by lipoxygenases, cyclooxygenases, and cytochrome P450 monooxygenases and are organized into subclasses: lipoxins (Lx), resolvins (Rv), protectins (PDs), and maresins (Mar) [33,34,35,36]. They work as the counterregulatory mediators of the natural resolution of inflammation, which is now recognized as an active host response, including asthmatic AI [37]. This signaling is impaired in asthma and in the resolution of asthmatic AI [37,38,39]. Synthetic SPMs have given promising results as potential counterinflammatory treatments in asthma models [37,38,39,40]; whether SPMs can dampen PM-induced AI has not been studied, but we found one report that LxA4 was therapeutic in PM-induced asthma [41], which led us to hypothesize that SPMs can ameliorate maternally transmitted asthma induced by prenatal particle exposure.

This paucity of experimentation stems from a lack of knowledge on the effects of PM2.5 on the SPM network—we found only one report on ultrafine PM effects [42]—a gap we helped to fill via a prior SPM-focused lipidomic profiling which detected a decline in a set of SPMs in the lungs of the neonates of exposed mothers [43]. Among the counterinflammatory SPMs seen declined were LxA4 and RvE2; hence, we hypothesized that therapeutic resupplementation of these molecules into the lungs can improve the resolution of allergic airway inflammation triggered by perinatal exposure to particles.

## 2. Results

**Figure 1:**
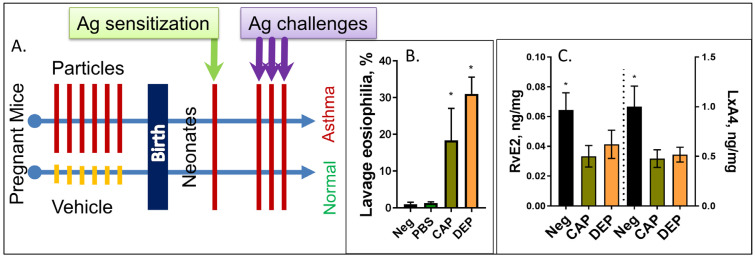
**Schematic of the study protocol.** (**A**) Pregnant dams at E14-E20 days of gestation received 6 intranasal instillations of CAP or DEP suspensions, or vehicle. After birth, the pups received a single i.p. sensitization injection with ovalbumin OVA at postnatal day P3 and a set of 3 OVA aerosol challenges at days P12-14, and were then analyzed at P16. Pups born to particle-exposed mothers but not control mothers developed asthma phenotype seen as eosinophilic AI (**B**) and other asthma-like features (airway hyperresponsiveness, lung tissue infiltration, and cytokine increases (not shown, see [26,27,28,29,30,31])); (n) = 12, and * *p* < 0.05. (**C**) Lipidomic profiling of the neonate lungs shows decreased levels of LxA4 and RvE2 in pups of CAP or DEP exposed mothers but not controls (reanalysis from [43]); (n) = 9, and * *p* < 0.05.

Maternal gestational exposure to CAPs or DEPs elicits an enhanced “preparedness” to respond to the low-dose single-i.p. allergen protocol with the asthma phenotype as published previously [26,27] and reconfirmed here for quality assurance (Figure 1B). We have also confirmed the decreased levels of LxA4 and RvE2 in the lungs of CAP and DEP progeny, which we identified during the profiling of lung tissue in [43]; the reanalyzed data are added here in Figure 1C.

**Figure 2:**
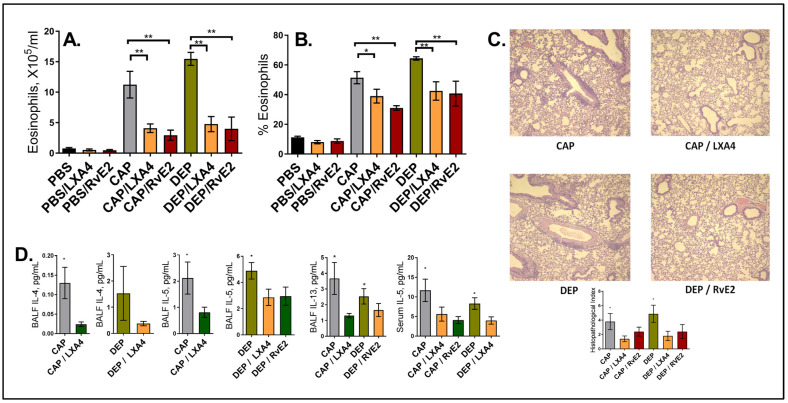
**LxA4’s and RvE2’s effect on resolution of AI after the low-dose allergen**. (**A**) BAL eosinophil counts, (**B**) percentage of eosinophils of total BAL cells, (**C**) lung histopathology representative images (H and E staining, ×100) and scoring, and (**D**) select cytokine measurements in BAL fluid and serum. Only 1 representative experiment of 3 is shown; (n) = 40. * *p* < 0.05, and ** *p* < 0.01.

Intra-airway treatment with LxA4 or RvE2 partly abrogated the maternal effect. The BAL eosinophil counts were reduced in the “at risk” offspring of the CAP or DEPexposed mothers by more than a half after either LxA4 or RvE2 treatment (Figure 2A), and the eosinophil percentages declined by ~ 20%; however, the eosinophils were not entirely eliminated and did not decline to the levels seen in the controls (Figure 2B). LxA4 and RvE2 had no effect on the offspring of the control PBS-exposed mothers that received the same low-dose OVA protocol, which was innocuous for them. The minimal airway eosinophilia in this group was not affected by LxA4 or RvE2 treatment. The lung tissue infiltration in the histopathology (Figure 2C) was consistent with the BAL eosinophilia findings. Again, there was no effect in the PBS group (not shown due to space constraints).

The BAL and serum cytokine levels in this model are usually consistent and directly correlate with BAL eosinophilia. Here, we were able to detect BAL IL-4, IL-5, and IL-13 in select samples: LxA4 treatment decreased BAL IL-4 in the offspring of the CAP and DEP exposed mice; LxA4 and RvE2 decreased the levels of BAL IL-5 in the DEP group, LxA4’s effect on BAL IL-13 was seen in the CAP offspring, RvE2’s effect on BAL IL-13 was seen in the DPE offspring, and serum IL-5 was decreased by LxA4 and RvE2 in the CAP group and by LxA4 in the DEP group (Figure 2D).

In combination, these data suggest that intra-airway treatment with LxA4 and RvE2 ameliorated but not entirely abrogated the asthma phenotype, which was seen as an improved resolution of the AI elicited by OVA in the offspring of both CAP and DEP exposed mothers.

## 3. Discussion

Maternal (more so than paternal) asthma [44,45] and maternal exposure to environmental offenders, including air pollution and cigarette smoke [46,47,48,49,50,51], are strongly linked to asthma in a child. Although the mechanism of this maternal effect remains undefined, one possibility is that the maternal immune system acts to influence the developing immune system of the child through epigenetic mechanisms [28,52]. These studies stem from the insightful “Barker Hypothesis” [53], which has sparked a growing appreciation of how maternal exposure triggers or aggravates disease later in life (“prenatal programming”). Our mouse model recapitulates the maternal asthma transmission seen in humans [28,29,30,31,54]. Moreover, maternal PM exposure had an effect similar to that of maternal asthma [26]. This PM effect transmitted not only to the F1 but also to the F2 and F3 generations after a single exposure [27].

This predisposition is seen after sensitization with a low dose of an allergen intentionally insufficient to produce the asthma phenotype in the control pups but causative of asthma in the “at-risk” pups. This model thus allows for the study of the pathogenic mechanisms of allergy origin that occur prior to encountering the sensitizing allergen, which is distinct from other allergen models.

The phenotype was evident in airway function (airway hyperresponsiveness in the methacholine test, obtained via direct resistance physiology and indirect plethysmography-based detection), lavage cytology (eosinophil counts), lung histopathology, and cytokine detection (IL-4, IL-5, IL-13, ELISA) [26,27,28,29,30,31,54]. These parameters usually tightly correlate, with BAL eosinophilia being the easiest and most accurate measure of the ‘strength’ of asthmatic AI. While PM does induce a neutrophil influx in the mothers (directly exposed to PM) [26], the neonatal response to OVA is a typical eosinophilic response and does not involve neutrophils. Maternal inflammation, thus, results in increased preparedness to OVA-induced asthma in the pups, seen as a typical eosinophilic response (because the pups are not directly exposed to PM). The maternal effect is allergen-independent, i.e., maternal OVA exposure leads to increased susceptibility to an unrelated allergen [54]. Importantly, we did not observe any increased susceptibility in the F1 pups of an OVA-allergic father mated to a normal mother, which focused our attention on the maternal lineage. The maternal effect could not be explained by the known mechanisms that mediate maternal phenomena: transplacental cytokines, mitochondrial DNA, or imprinting. This prompted us to look at previously unrecognized mechanisms, including the SPM network.

Here, we employed two types of multicomponent particles that are highly relevant and representative of real-world PM problems. Concentrated urban air particles (CAPs) model the exposure of a city dweller and serve as a helpful surrogate for urban air pollutants [55]. Human exposure to CAPs is ubiquitous in the urban setting and has been associated with the worsening of asthma symptoms [13,56]. CAPs, like all aerosolized particles, in the airways are scavenged by macrophages [57] and dendritic cells [58] and elicit proinflammatory responses [59]; CAPs comprise a variety of compositions and produce a range of deleterious effects, although they always include inflammation [60]. Coexposure to CAPs significantly worsens allergen-induced AI [13,60,61].

Diesel exhaust particles (DEPs) are known to be proinflammatory and proallergic [17], possibly due to their soluble components, including pyrene, a known stimulant of IL-4 expression [62]. Human exposure occurs through the exhaust of diesel engines and is postulated to be a component of the higher asthma risk in high-traffic areas [18,63]. The significance of DEPs is highlighted by the recent “dieselgate” scandal, which brought to light that real-life human exposure to diesel fumes is several times greater than theoretically thought [64,65,66].

Based on prior work stating that CAPs and DEPs impair the ability to elicit the production of several SPMs [43], we speculated that the SPM network may mediate the maternal “asthma preparedness” effect of the particles, which would render a new mechanistic insight into our understanding of how particle pollution contributes to asthma and may open novel therapeutic avenues. There is a great unmet need for novel therapeutic strategies in asthma [67]. The emerging SPM treatments are promising in asthmatic [68,69] and other chronic inflammation [70]: LxB4 promoted the resolution of AI in the airway [71], RvD3 treatment mitigated murine peritonitis and dermal inflammation [72], treatment with Mar1 was protective in a model of skin inflammation [73], and PD1 dampened AI and hyperresponsiveness [74]. This therapeutic direction is exciting because SPMs are “immunoresolvents”, not immunosuppressants, and, thus, may be able to ameliorate AI without affecting normal defensive immunity [75]. SPMs act through specific receptors [76,77]: specifically, LxA4 binds to the ALX/FPR2 receptor [78], and RvE1 and E2 both act via the ERV1 (CMKLR1) and BLT1 receptors [78,79]. This signaling leads to a number of counterinflammatory proresolving events that are yet to be fully elucidated.

Our data indicate that either LxA4 or RvE2 intra-airway (intranasal) treatment substantially diminished the BAL eosinophil counts in the offspring of both the CAP- and DEP-exposed mothers vs. the vehicle control and that it ameliorated lung tissue infiltration as seen in Figure 2A–C. The SPMs had no effect in the noninflamed control pups born to PBS exposed mothers. Notably, although the decrease in the eosinophil counts was substantial, the SPMs did not completely abrogate eosinophilia. This could be due to the doses and route used or could be a limitation of the power of SPMs, which will be determined in future studies. Our cytokine ELISA measurements were technically challenged by the small yields of the lavage and serum that could be obtained from the neonate pups and the relatively low level of cytokine induction. We were able to detect a statistically significant decline in BAL IL-4, IL-5, and IL-13 after LxA4 in the CAP model and after RvE2 in the DEP model, and serum IL-5 was decreased in both models; however, we did not register a decline in all the cytokines in both models as anticipated, which we attribute to technical difficulties. Nevertheless, the data are consistent with the other results of the model and support the beneficial therapeutic effect of LxA4 or RvE2 on the landscape of proallergic cytokines.

To put our findings into context: in [41], LxA4 ameliorated asthma in directly exposed adult mice, where PM2.5 was given simultaneously with OVA as an adjuvant that aggravates the effect. In contrast, in our model, the PM serves as a maternal trigger; hence, the newborns were not directly exposed to PM. LxA4 has also been successful in nonparticle allergen-only asthma [80]. Other lipoxins have shown therapeutic effects against asthma in mice as well [71].

To our knowledge, resolvins have not been tested in the context of PM-induced effects, but RvD1 and E1 have been shown to ameliorate experimental asthma in allergen models [81,82,83]; we were not able to find reports on RvE2 treatment in such models. Our report is also the first to demonstrate the therapeutic effect of SPMs in maternally transmitted asthma.

Conclusion. Intra-airway treatment with synthetic LxA4 or RvE2 mitigated the OVA-induced asthma phenotype in the neonates born to mothers exposed to CAPs and DEPs, seen as a reduction in BAL eosinophilia, lung tissue inflammatory infiltration, and proallergic cytokine levels. This suggests that SPM therapies may be able to mitigate hazardous environmental exposure that triggers asthma.

## 4. Materials and Methods

### 4.1. Animals

Time-pregnant (E13) BALB/c mice were obtained from Charles River Laboratories (Wilmington, MA, USA). They were caged individually in the specific pathogen-free (SPF) barrier facility of Rhode Island Hospital. They were maintained at 22–24 °C with a 12 h dark and 12 h light cycle and were fed a commercial pelleted diet and water ad libitum.

### 4.2. Particles

The concentrated urban air particles (CAPs) were obtained from Boston City air using Harvard Ambient Particle Concentrator [84,85] and are well characterized [16,26,27,86,87].

Diesel exhaust particles (DEPs), CAS Number 1333-86-4, were generously provided by Dr. Ian Gilmour from the U.S. Environmental Protection Agency and were used in earlier studies [17,26,27,43]. All particles were of comparable “fine” size of the PM2.5 class with mean particle size of ~1 μm, although they were not identical (see micrographs in [88]).

### 4.3. Exposure

We administered CAP or DEP suspensions in sterile LPS-free PBS (Lonza, Walkersville, MD, USA) solution through intranasal insufflations at a dose of 8.6 µg/mouse in 50 µL PBS. Particle samples were baked at 165 °C for 3 h to eliminate endotoxins, were aliquoted, and were stored frozen at −80 °C. Before instillation, the particles were freshly sonicated on ice to break up clumps and to assure a homogenous suspension using Qsonica Q55 probe sonicator. Briefly, after light isoflurane anesthesia, a droplet (25 µL) was placed on the nares and was inspired by the mouse followed by another 25 µL volume droplet. Exposure was performed 1 time a day for 6 days at E14-E20 days of gestation. Dams were allowed to give birth; their newborns received a single intraperitoneal (i.p.) sensitization injection of 50 µg ovalbumin (OVA) + aluminum hydroxide adjuvant (alum) at postnatal day P3 and a set of 3 daily 7 min 1% OVA challenges (grade V; Sigma–Aldrich, St. Louis, MO, USA) in PBS 2 weeks later. The aerosol exposure was performed within individual compartments of a mouse pie chamber (Braintree Scientific, Braintree, MA, USA) using a Pari IS2 nebulizer (Sun Medical Supply, Henderson, NC, USA) connected to an air compressor (Pulmo-Aide; DeVilbiss). This “low-dose OVA protocol” remained innocuous in control pups (which was in contrast to the 2X i.p. protocol commonly used elsewhere) but elicited asthma-like phenotype in the offspring of particle-exposed mothers [26,27,28,29,30,31,54], and it served to test how exposure to particulates predisposes one to asthma. The protocol is summarized in Figure 1.

### 4.4. Pathologic Analysis

At 24 h after the last aerosol, the newborns were euthanized with sodium pentobarbital. BAL followed a standard procedure of 5 times × 300 µL washes with PBS [26,27,28,29,30,31]. After centrifugation at 1200 RPM (300 G) for 10 min, the fluid was stored for cytokine assays. The pellet was resuspended in 100 µL PBS; BAL differential cell counts were performed on cytocentrifuge slides (Cytospin 2; Shandon, Pittsburgh, PA, USA), including enumeration of the percentage of eosinophils out of total BAL cells. After lavage, the lungs were fixed with 10% buffered formalin. After paraffin embedding, sections for microscopy were stained with hematoxylin and eosin (H and E). Slides were scored by a blind observer for severity (score of 1 for 1–3 cells thick, 2 for 4–10 cells thick, and 3 for >10 cells thick) and extent (score of 1 for <25%, 2 for <50%, and 3 for >50% coverage) of inflammatory infiltration, and the inflammatory index was calculated as “severity” × “extent” [26,27,28,29,30,31]. Blood was collected through intracardiac puncture. Levels of cytokines in BAL fluid or serum were measured via ELISA (R&D Systems, Minneapolis, MN, USA).

### 4.5. SPMs

Synthetic LxA4 (#90410) and RvE2 (#13827) were obtained from Cayman Chemical (Ann Arbor, MI, USA) and were dissolved in PBS. They were administrated via intranasal instillations of 750 ng/mouse LxA4 or 300 ng/mouse RvE2 once daily for 3 days immediately following OVA aerosols.

All studies complied with the ARRIVE guidelines, were performed in compliance with the National Institutes of Health’s guide for the care and use of laboratory animals, and were approved by the IACUC of Rhode Island Hospital.

### 4.6. Data Analysis

To examine the significance of differences, we performed ANOVA with the Newman–Keuls post hoc test or Fisher’s LSD test. Differences were considered significant when *p* < 0.05. We focused on comparisons in which a mean in a treatment group (LXA4 or RvE2) was significantly different from either control (CAPs or DEPs), and these are indicated with an asterisk on the charts.

## Data Availability

Data may be made available by the principal investigator, A.V.F., in response to a reasonable request through the journal’s editorial office.
